# A redetermination of 2-(6-diethyl­amino-3-diethyl­iminio-3*H*-xanthen-9-yl)benzoate–ethyl gallate (1/1) at room temperature

**DOI:** 10.1107/S160053680900751X

**Published:** 2009-03-06

**Authors:** Jin Mizuguchi, Kazuyuki Sato

**Affiliations:** a Department of Applied Physics, Graduate School of Engineering, Yokohama National University, Tokiwadai 79-5, Hodogaya-ku, Yokohama 240-8501, Japan

## Abstract

The title compound, C_28_H_30_N_2_O_3_·C_9_H_10_O_5_, is a well known red leuco complex of 2-(6-diethyl­amino-3-diethyl­iminio-3*H*-xanthene-9-yl)benzoate (rhodamine B base abbreviated to RBB: leuco dye) with ethyl gallate (EG: developer). The structure of the complex at room temperature has recently been reported by Sekiguchi, Takayama, Gotanda & Sano [(2007) *Chem. Lett.* 36, 1010–1011]. The RBB–EG complex forms a dimer (RBB⋯EG⋯EG⋯RBB) through inter­molecular O—H⋯O hydrogen bonds. In a subsequent re-examination of the structure at room temperature, we found the RBB mol­ecule to be disordered with a methyl group of one ethyl substituent of a diethyl­amino group at one extremity of the xanthene unit disordered over two positions [occupancies: 0.735 (5)/0.265 (5)]. Furthermore, at the other end of the xanthene residue, the entire diethyl­amino substituent (*i.e.* the N atom and the associated C and H atoms) was also disordered over two sites with occupancies 0.653 (7)/0.347 (7). This leads to four kinds of RBB conformations, which, in turn, results in the formation of 16 discrete RBB⋯EG⋯EG⋯RBB dimers in the crystal.

## Related literature

For the previous determination of the structure of the 1:1 RBB/EG complex at room temperature, see: Sekiguchi *et al.* (2007 ▶) and for the structure of a second triclinic form of the same complex at 93 K, see: Mizuguchi (2008 ▶). For the related structure of *n*-propyl gallate, see: Iwata *et al.* (2005 ▶); Hitachi *et al.* (2005 ▶).
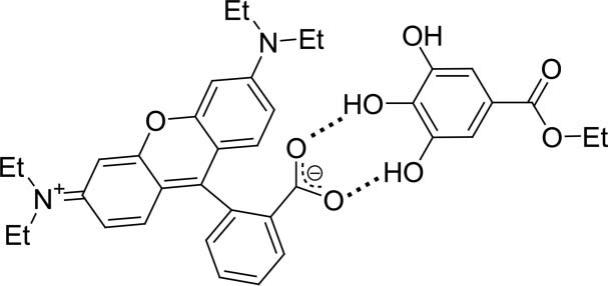


## Experimental

### 

#### Crystal data

C_28_H_30_N_2_O_3_·C_9_H_10_O_5_
*M* *_r_* = 640.71
Triclinic, 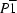

*a* = 11.4721 (3) Å
*b* = 11.8036 (3) Å
*c* = 12.4816 (3) Å
α = 85.805 (2)°
β = 87.202 (1)°
γ = 81.973 (1)°
*V* = 1667.84 (7) Å^3^
*Z* = 2
Cu *K*α radiation
μ = 0.74 mm^−1^
*T* = 296 K
0.20 × 0.20 × 0.20 mm

#### Data collection

Rigaku R-AXIS RAPID diffractometer
Absorption correction: multi-scan (Higashi, 1995 ▶) *T*_min_ = 0.851, *T*_max_ = 0.863
15046 measured reflections
5610 independent reflections
3355 reflections with *F* ^2^ > 2σ(*F* ^2^)
*R*_int_ = 0.077

#### Refinement

*R*[*F* ^2^ > 2σ(*F* ^2^)] = 0.050
*wR*(*F* ^2^) = 0.141
*S* = 0.93
5610 reflections
476 parameters
31 restraints
H-atom parameters constrained
Δρ_max_ = 0.16 e Å^−3^
Δρ_min_ = −0.19 e Å^−3^

### 

Data collection: *PROCESS-AUTO* (Rigaku, 1998 ▶); cell refinement: *PROCESS-AUTO*; data reduction: *CrystalStructure* (Rigaku/MSC & Rigaku, 2006 ▶); program(s) used to solve structure: *SIR2004* (Burla *et al*., 2005 ▶); program(s) used to refine structure: *SHELXL97* (Sheldrick, 2008 ▶); molecular graphics: *ORTEPIII* (Burnett & Johnson, 1996 ▶); software used to prepare material for publication: *CrystalStructure*.

## Supplementary Material

Crystal structure: contains datablocks global, I. DOI: 10.1107/S160053680900751X/sj2586sup1.cif

Structure factors: contains datablocks I. DOI: 10.1107/S160053680900751X/sj2586Isup2.hkl

Additional supplementary materials:  crystallographic information; 3D view; checkCIF report

## Figures and Tables

**Table 1:** Hydrogen-bond geometry (Å, °)

*D*—H⋯*A*	*D*—H	H⋯*A*	*D*⋯*A*	*D*—H⋯*A*
O4—H4*O*⋯O7^i^	0.82	2.00	2.811 (2)	168
O5—H5*O*⋯O2	0.82	2.79	3.257 (2)	118
O5—H5*O*⋯O3	0.82	1.78	2.579 (2)	164
O6—H6*O*⋯O2	0.82	1.86	2.5991 (18)	148
O6—H6*O*⋯O3	0.82	2.78	3.3842 (19)	132
O6—H6*O*⋯O5	0.82	2.47	2.8758 (19)	112

Symmetry code: (i) 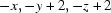.

**Fig. 1.**
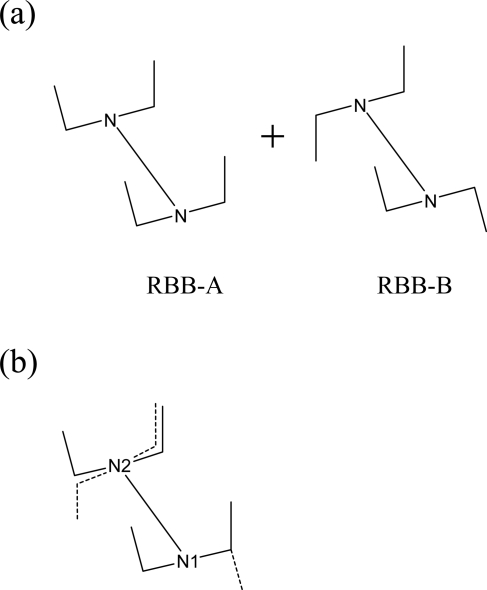
(a) A schematic representation of the two independent conformations of RBB found at 93 K, where RBB-A illustrates the diethylamino substituents on the same side while in RBB-B they are on opposite sides, of the xanthene plane. (b) The disordered structure of the title molecule at room temperature, where the major and minor disorder components are depicted as solid and dotted lines, respectively.

**Fig. 2.**
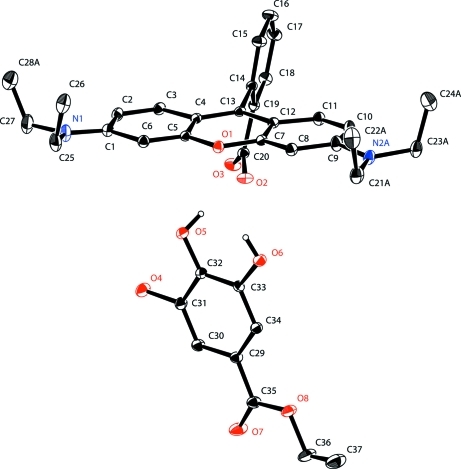
Plot of one of the four possible disordered conformations of (I), showing 10% displacement ellipsoids. Hydrogen atoms except for those involved in the intermolecular hydrogen bonds are omitted for clarity. The "major/major" combination shown here corresponds to the RBB-A form found at 93K and shown in Fig. 1a.

**Fig. 3.**
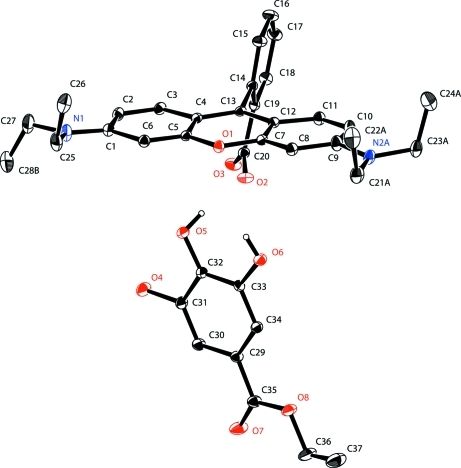
Plot of one of the four possible disordered conformations of (I), showing 10% displacement ellipsoids. Hydrogen atoms except for those involved in the intermolecular hydrogen bonds are omitted for clarity.

**Fig. 4.**
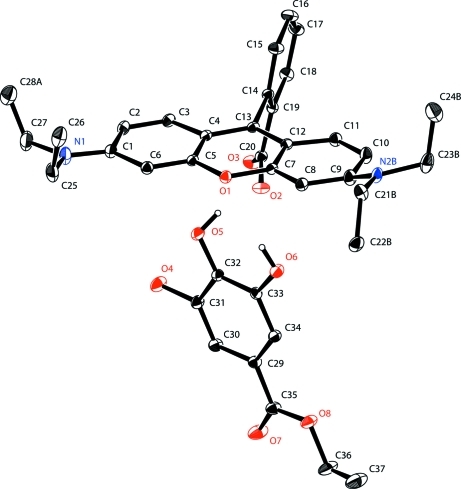
Plot of one of the four possible disordered conformations of (I), showing 10% displacement ellipsoids. Hydrogen atoms except for those involved in the intermolecular hydrogen bonds are omitted for clarity.

**Fig. 5.**
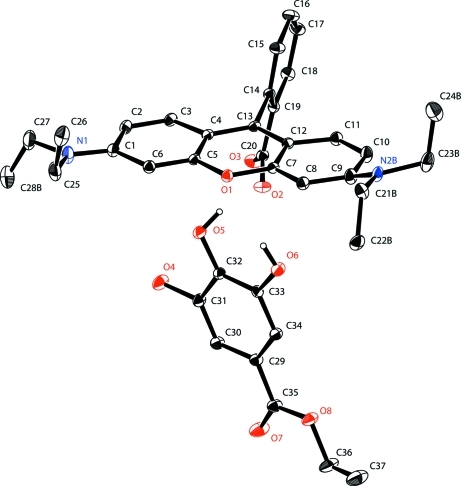
Plot of one of the four possible disordered conformations of (I), showing 10% displacement ellipsoids. Hydrogen atoms except for those involved in the intermolecular hydrogen bonds are omitted for clarity. The "minor/minor" combination shown here corresponds to the RBB-B form found at 93K and shown in Fig. 1a.

**Fig. 6.**
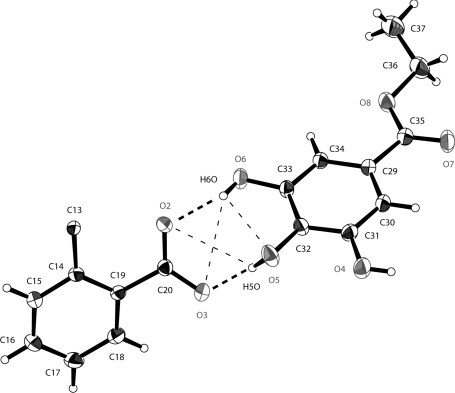
Intra- and intermolecular O—H···O hydrogen bonds leading to the formation of the RBB/EG complex. For clarity, only the C14/C19 benzene ring and its substituents are shown for the RBB unit.

**Fig. 7.**
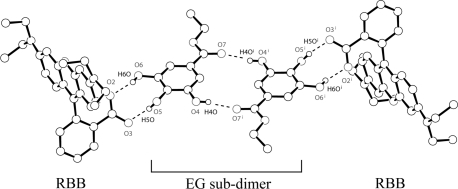
The hydrogen-bonded RBB (major)···EG···EG···RBB (major) dimer formed by linking two RBB/EG complexes through intermolecular O—H···O hydrogen bonds.

## supplementary crystallographic information

### Comment

The title compound, C_28_H_30_N_2_O_3_.C_9_H_10_O_5_, is a well known red leuco complex of 2-(6-diethylamino-3-diethyliminio-3*H*-xanthene-9-yl)benzoate with ethyl gallate (rhodamine B base abbreviated to RBB: leuco dye) with ethyl gallate (EG: developer). The structure of the RBB/EG complex at room temperature has recently been reported by Sekiguchi *et al.* (2007), where the RBB conformation is uniquely RBB-A as shown schematically in Fig. 1*a*. That is, the ethyl groups of the xanthene diethylamino substituents lie on the same side of the xanthene plane in RBB-A. Quite recently, we have also found a new triclinic phase with two discrete base/developer complexes (RBB-A/EG-A and RBB-B/EG-B: see Fig. 1*a*) at 93 K (Mizuguchi, 2008). In both phases, two RBBs are connected by a sub-dimer of EG through intermolecular O—H···O hydrogen bonds. However, close inspection of the supplementary CIF of the report of Sekiguchi *et al.* (2007) revealed that there was a residual electron density peak of about 1.35 e Å^-3^. For this reason, a redetermination of the structure has been carried out at room temperature in the present investigation. This revealed that the RBB molecule is disordered as shown in Fig. 1*b* with the C28 methyl group of one ethyl substituent of the N1 diethylamino group at one extremity of the xanthene moiety as well as the entire diethylamino-substituent (*i.e.* N2 atom with the associated C and H atoms) at the other end of the xanthene unit disordered over two positions.

As shown in Fig. 1*b*, the RBB molecule is disordered at C28A/C28B together with their associated H atoms at one extremity of the xanthene moiety. The disordered structure is separated into the major (0.735 (5)) and minor (0.265 (5)) components which correspond to the RBB-A and RBB-B forms respectively, as shown in Fig. 1*a*. These are similar to those found at 93K, where the diethylamino-substituents lie either on the same side, or on opposite sides of the xanthene plane. Similarly, the disorder at the entire N2 diethylamino-substituents at the other end of the xanthene plane also leads to the presence of the two conformations described above: N2A group (diethylamino-substituents on the same side; occupancy 0.653 (7)) and N2B group (on opposite sides; occupancy 0.347 (7)). Figs. 2–5 show plots of the four possible structures of (I). Of these, the previous report (Sekiguchi *et al.*, 2007) only identified the conformation shown in Fig. 2.

The lactone ring is opened to form a zwitterionic structure and the benzene ring with the carboxylate is twisted to be nearly perpendicular to the xanthene plane with a dihedral angle of 98.9 (1)° between the O1/C1—C13 plane of the xanthene and the C14—C19 plane of the benzene ring. The xanthene moiety is nearly flat (mean deviation from the least-squares plane, 0.0300 Å).

There are intra and intermolecular O—H···O hydrogen bonds leading to the formation of the RBB/EG complexes as shown in Fig. 6. For example, two major RBB/EG complexes are further connected by intermolecular O—H···O hydrogen bonds between two EGs to form a dimer as shown in Fig. 7. The existence of the four possible RBB conformations leads to the formation of 16 kinds of RBB···EG···EG···RBB dimers in the crystal. The formation of the EG dimer is similar to that found in *n*-propyl gallate (Iwata *et al.*, 2005; Hitachi *et al.*, 2005).

### Experimental

Rhodamine B base and 4-hydroxybenzophenone were purchased from Sigma-Aldrich Corp. and Wako Pure Chemical Industries, Ltd., respectively. Single crystals of (I) were grown by recrystallization from a toluene solution which includes an equimolar quantity of both chemicals. After 24 h, a number of red crystals were obtained in the form of blocks.

### Refinement

The C28A and C28B methyl groups were disordered over two positions with occupancies of 0.735 (5)/0.265 (5), respectively.  Also the disorder at N2 (*i.e.*  N2A/N2B) extends to C23–C22–N2–C23–C24 and the associated H atoms. The occupancies for the N2A and N2B groups and their associated atoms are 0.653 (7) and 0.347 (7), respectively. All H atoms were placed in geometrically idealized position and constrained to ride on their parent atoms, with C—H = 0.93, 0.96, and 0.97 Å, and *U*_iso_(H) = 1.2 and 1.5 *U*_eq_(C), respectively, and with O—H = 0.82 Å and *U*_iso_(H) = 1.2.

### Crystal data

**Table:**

C_28_H_30_N_2_O_3_·C_9_H_10_O_5_	*Z* = 2
*M**_r_* = 640.71	*F*(000) = 680.00
Triclinic, *P*1	*D*_x_ = 1.276 Mg m^−^^3^
Hall symbol: -P 1	Cu *K*α radiation, λ = 1.54187 Å
*a* = 11.4721 (3) Å	Cell parameters from 10532 reflections
*b* = 11.8036 (3) Å	θ = 3.0–68.5°
*c* = 12.4816 (3) Å	µ = 0.74 mm^−^^1^
α = 85.805 (2)°	*T* = 296 K
β = 87.202 (1)°	Block, red
γ = 81.973 (1)°	0.20 × 0.20 × 0.20 mm
*V* = 1667.84 (7) Å^3^	

### Data collection

**Table:**

Rigaku R-AXIS RAPID diffractometer	3355 reflections with *F*^2^ > 2σ(*F*^2^)
ω scans	*R*_int_ = 0.077
Absorption correction: multi-scan (Higashi, 1995)	θ_max_ = 68.2°
*T*_min_ = 0.851, *T*_max_ = 0.863	*h* = −13→13
15046 measured reflections	*k* = −13→14
5610 independent reflections	*l* = −14→15

### Refinement

**Table:**

Refinement on *F*^2^	31 restraints
*R*[*F*^2^ > 2σ(*F*^2^)] = 0.050	H-atom parameters constrained
*wR*(*F*^2^) = 0.141	*w* = 1/[σ^2^(*F*_o_^2^) + (0.0812*P*)^2^] where *P* = (*F*_o_^2^ + 2*F*_c_^2^)/3
*S* = 0.93	(Δ/σ)_max_ < 0.001
5610 reflections	Δρ_max_ = 0.16 e Å^−^^3^
476 parameters	Δρ_min_ = −0.19 e Å^−^^3^

### Special details

**Table:**

Geometry. All e.s.d.'s (except the e.s.d. in the dihedral angle between two l.s. planes) are estimated using the full covariance matrix. The cell e.s.d.'s are taken into account individually in the estimation of e.s.d.'s in distances, angles and torsion angles; correlations between e.s.d.'s in cell parameters are only used when they are defined by crystal symmetry. An approximate (isotropic) treatment of cell e.s.d.'s is used for estimating e.s.d.'s involving l.s. planes.
Refinement. Refinement of *F*^2^ against ALL reflections. The weighted *R*-factor *wR* and goodness of fit *S* are based on *F*^2^, conventional *R*-factors *R* are based on *F*, with *F* set to zero for negative *F*^2^. The threshold expression of *F*^2^ > σ(*F*^2^) is used only for calculating *R*-factors(gt) *etc*. and is not relevant to the choice of reflections for refinement. *R*-factors based on *F*^2^ are statistically about twice as large as those based on *F*, and *R*- factors based on ALL data will be even larger.

### Fractional atomic coordinates and isotropic or equivalent isotropic displacement parameters (Å^2^)

**Table:**

	*x*	*y*	*z*	*U*_iso_*/*U*_eq_	Occ. (<1)
O1	0.62196 (11)	0.41220 (11)	0.45212 (10)	0.0516 (3)	
O2	0.57084 (12)	0.69798 (13)	0.66780 (12)	0.0664 (4)	
O3	0.60107 (12)	0.76396 (14)	0.82411 (12)	0.0728 (5)	
O4	0.21545 (15)	0.78684 (15)	1.01780 (12)	0.0889 (6)	
H4O	0.1650	0.8183	1.0587	0.107*	
O5	0.38741 (12)	0.72981 (13)	0.87434 (12)	0.0768 (5)	
H5O	0.4509	0.7531	0.8609	0.092*	
O6	0.37281 (12)	0.83482 (12)	0.65926 (11)	0.0693 (5)	
H6O	0.4205	0.7806	0.6808	0.083*	
O7	−0.06957 (15)	1.09897 (17)	0.82505 (14)	0.1034 (7)	
O8	0.01167 (13)	1.10673 (14)	0.66131 (13)	0.0808 (5)	
N1	0.54396 (19)	0.14504 (17)	0.73818 (16)	0.0812 (6)	
C1	0.58390 (19)	0.24391 (19)	0.70248 (18)	0.0604 (6)	
C2	0.63447 (19)	0.30991 (18)	0.77375 (17)	0.0611 (6)	
H2	0.6350	0.2872	0.8467	0.073*	
C3	0.68183 (18)	0.40497 (18)	0.73815 (16)	0.0553 (5)	
H3	0.7149	0.4456	0.7872	0.066*	
C4	0.68265 (16)	0.44474 (16)	0.62802 (15)	0.0462 (5)	
C5	0.62725 (16)	0.38095 (16)	0.55992 (15)	0.0463 (5)	
C6	0.58048 (17)	0.28344 (18)	0.59322 (17)	0.0556 (5)	
H6	0.5466	0.2435	0.5442	0.067*	
C7	0.67239 (16)	0.50429 (16)	0.40937 (16)	0.0468 (5)	
C8	0.66879 (18)	0.52327 (18)	0.29939 (16)	0.0559 (5)	
H8	0.6337	0.4751	0.2590	0.067*	
C9	0.7186 (2)	0.6162 (2)	0.24908 (18)	0.0715 (7)	
C10	0.7693 (2)	0.6883 (2)	0.31501 (18)	0.0724 (7)	
H10	0.7999	0.7523	0.2835	0.087*	
C11	0.77389 (19)	0.66561 (19)	0.42244 (17)	0.0598 (6)	
H11	0.8080	0.7143	0.4630	0.072*	
C12	0.72836 (16)	0.57016 (16)	0.47512 (15)	0.0459 (5)	
C13	0.73447 (15)	0.53923 (16)	0.58585 (15)	0.0442 (5)	
C14	0.81034 (16)	0.59702 (16)	0.65346 (15)	0.0470 (5)	
C15	0.93057 (18)	0.5612 (2)	0.64624 (18)	0.0638 (6)	
H15	0.9598	0.5038	0.6009	0.077*	
C16	1.0074 (2)	0.6089 (2)	0.7050 (2)	0.0762 (7)	
H16	1.0877	0.5830	0.7000	0.091*	
C17	0.9652 (2)	0.6951 (2)	0.7711 (2)	0.0738 (7)	
H17	1.0170	0.7301	0.8085	0.089*	
C18	0.84517 (19)	0.72899 (19)	0.78127 (18)	0.0610 (6)	
H18	0.8165	0.7854	0.8278	0.073*	
C19	0.76644 (16)	0.68071 (16)	0.72356 (15)	0.0456 (5)	
C20	0.63599 (17)	0.71751 (16)	0.73885 (17)	0.0507 (5)	
N2A	0.7055 (4)	0.6508 (4)	0.1419 (3)	0.0667 (12)	0.653 (7)
C21A	0.6481 (6)	0.5815 (5)	0.0733 (4)	0.0907 (19)	0.653 (7)
H21A	0.6120	0.6300	0.0144	0.109*	0.653 (7)
H21B	0.5865	0.5474	0.1147	0.109*	0.653 (7)
C22A	0.7368 (7)	0.4881 (7)	0.0290 (6)	0.121 (3)	0.653 (7)
H22A	0.6985	0.4446	−0.0173	0.181*	0.653 (7)
H22B	0.7701	0.4383	0.0872	0.181*	0.653 (7)
H22C	0.7982	0.5218	−0.0112	0.181*	0.653 (7)
C23A	0.7631 (5)	0.7444 (5)	0.0886 (5)	0.0845 (18)	0.653 (7)
H23A	0.7451	0.8113	0.1302	0.101*	0.653 (7)
H23B	0.7306	0.7641	0.0183	0.101*	0.653 (7)
C24A	0.8941 (6)	0.7159 (9)	0.0753 (6)	0.119 (3)	0.653 (7)
H24A	0.9263	0.7806	0.0405	0.179*	0.653 (7)
H24B	0.9128	0.6512	0.0322	0.179*	0.653 (7)
H24C	0.9273	0.6976	0.1446	0.179*	0.653 (7)
N2B	0.7578 (9)	0.6057 (7)	0.1399 (5)	0.067 (2)	0.347 (7)
C21B	0.7208 (8)	0.5243 (11)	0.0689 (8)	0.070 (3)	0.347 (7)
H21C	0.7341	0.4489	0.1063	0.084*	0.347 (7)
H21D	0.7742	0.5225	0.0062	0.084*	0.347 (7)
C22B	0.5970 (7)	0.5395 (8)	0.0285 (7)	0.083 (3)	0.347 (7)
H22D	0.5889	0.4783	−0.0160	0.124*	0.347 (7)
H22E	0.5821	0.6116	−0.0127	0.124*	0.347 (7)
H22F	0.5415	0.5383	0.0885	0.124*	0.347 (7)
C23B	0.8235 (11)	0.6926 (10)	0.0826 (10)	0.097 (4)	0.347 (7)
H23C	0.8078	0.7636	0.1186	0.116*	0.347 (7)
H23D	0.7968	0.7078	0.0097	0.116*	0.347 (7)
C24B	0.9536 (10)	0.6518 (13)	0.0797 (12)	0.119 (3)	0.347 (7)
H24D	0.9947	0.7101	0.0439	0.179*	0.347 (7)
H24E	0.9695	0.5832	0.0416	0.179*	0.347 (7)
H24F	0.9798	0.6360	0.1518	0.179*	0.347 (7)
C25	0.5100 (3)	0.0666 (2)	0.6627 (3)	0.0976 (9)	
H25A	0.4601	0.1105	0.6096	0.117*	
H25B	0.4642	0.0128	0.7018	0.117*	
C26	0.6126 (3)	0.0012 (2)	0.6064 (2)	0.1088 (10)	
H26A	0.5863	−0.0588	0.5697	0.163*	
H26B	0.6693	−0.0318	0.6579	0.163*	
H26C	0.6482	0.0519	0.5552	0.163*	
C27	0.5405 (3)	0.1044 (3)	0.8530 (2)	0.1106 (11)	
H27A	0.5321	0.1708	0.8956	0.133*	0.735 (5)
H27B	0.4704	0.0674	0.8674	0.133*	0.735 (5)
H27C	0.5688	0.0229	0.8592	0.133*	0.265 (5)
H27D	0.5939	0.1429	0.8907	0.133*	0.265 (5)
C28A	0.6391 (4)	0.0270 (3)	0.8895 (4)	0.1142 (17)	0.735 (5)
H28A	0.6298	0.0102	0.9656	0.171*	0.735 (5)
H28B	0.7098	0.0612	0.8741	0.171*	0.735 (5)
H28C	0.6443	−0.0427	0.8533	0.171*	0.735 (5)
C28B	0.4273 (9)	0.1232 (10)	0.9041 (10)	0.122 (4)	0.265 (5)
H28D	0.4288	0.0834	0.9740	0.183*	0.265 (5)
H28E	0.3713	0.0951	0.8616	0.183*	0.265 (5)
H28F	0.4050	0.2038	0.9113	0.183*	0.265 (5)
C29	0.11073 (17)	0.97922 (17)	0.78864 (17)	0.0535 (5)	
C30	0.11491 (18)	0.92607 (19)	0.89144 (17)	0.0615 (6)	
H30	0.0550	0.9460	0.9423	0.074*	
C31	0.20825 (19)	0.84344 (19)	0.91795 (17)	0.0594 (6)	
C32	0.29921 (17)	0.81247 (17)	0.84399 (17)	0.0535 (5)	
C33	0.29226 (16)	0.86332 (17)	0.73917 (16)	0.0507 (5)	
C34	0.19940 (16)	0.94763 (17)	0.71342 (16)	0.0533 (5)	
H34	0.1967	0.9834	0.6445	0.064*	
C35	0.00929 (19)	1.0669 (2)	0.76249 (19)	0.0658 (6)	
C36	−0.0881 (2)	1.1878 (3)	0.6247 (2)	0.1044 (10)	
H36A	−0.1612	1.1623	0.6524	0.125*	
H36B	−0.0840	1.2626	0.6506	0.125*	
C37	−0.0845 (3)	1.1951 (3)	0.5094 (3)	0.1308 (13)	
H37A	−0.1495	1.2489	0.4839	0.196*	
H37B	−0.0898	1.1210	0.4844	0.196*	
H37C	−0.0119	1.2202	0.4826	0.196*	

### Atomic displacement parameters (Å^2^)

**Table:**

	*U*^11^	*U*^22^	*U*^33^	*U*^12^	*U*^13^	*U*^23^
O1	0.0572 (8)	0.0526 (8)	0.0461 (8)	−0.0103 (6)	−0.0067 (6)	−0.0016 (6)
O2	0.0483 (8)	0.0812 (11)	0.0693 (10)	0.0075 (7)	−0.0122 (7)	−0.0277 (8)
O3	0.0586 (9)	0.0995 (12)	0.0594 (10)	0.0023 (8)	0.0045 (7)	−0.0285 (9)
O4	0.0873 (11)	0.1121 (14)	0.0514 (10)	0.0240 (10)	0.0129 (8)	0.0185 (9)
O5	0.0579 (9)	0.0789 (11)	0.0819 (12)	0.0115 (8)	0.0084 (8)	0.0248 (9)
O6	0.0639 (9)	0.0755 (10)	0.0580 (9)	0.0156 (8)	0.0159 (7)	0.0057 (8)
O7	0.0816 (12)	0.1408 (17)	0.0679 (12)	0.0465 (11)	0.0147 (10)	−0.0040 (11)
O8	0.0677 (10)	0.0974 (12)	0.0635 (10)	0.0285 (9)	0.0027 (8)	0.0085 (9)
N1	0.1076 (16)	0.0714 (13)	0.0693 (14)	−0.0378 (12)	−0.0053 (12)	0.0142 (11)
C1	0.0624 (13)	0.0581 (14)	0.0593 (14)	−0.0089 (11)	0.0006 (11)	0.0053 (11)
C2	0.0745 (14)	0.0618 (14)	0.0446 (13)	−0.0062 (11)	−0.0027 (11)	0.0065 (11)
C3	0.0637 (13)	0.0564 (13)	0.0442 (12)	−0.0016 (10)	−0.0041 (10)	−0.0028 (10)
C4	0.0453 (10)	0.0487 (11)	0.0422 (11)	0.0017 (9)	−0.0034 (9)	−0.0007 (9)
C5	0.0455 (10)	0.0500 (12)	0.0407 (11)	0.0009 (9)	−0.0009 (8)	0.0000 (9)
C6	0.0595 (12)	0.0565 (13)	0.0514 (13)	−0.0103 (10)	−0.0053 (10)	−0.0006 (10)
C7	0.0456 (11)	0.0470 (11)	0.0462 (12)	−0.0011 (9)	−0.0038 (9)	−0.0017 (9)
C8	0.0634 (13)	0.0644 (14)	0.0414 (12)	−0.0134 (11)	−0.0086 (10)	−0.0015 (10)
C9	0.0893 (17)	0.0864 (17)	0.0431 (13)	−0.0308 (14)	−0.0096 (12)	0.0078 (12)
C10	0.0964 (18)	0.0740 (16)	0.0514 (14)	−0.0331 (14)	−0.0072 (13)	0.0085 (12)
C11	0.0689 (14)	0.0618 (14)	0.0512 (13)	−0.0166 (11)	−0.0084 (11)	−0.0010 (11)
C12	0.0472 (10)	0.0467 (11)	0.0435 (11)	−0.0035 (9)	−0.0044 (9)	−0.0046 (9)
C13	0.0432 (10)	0.0481 (11)	0.0386 (10)	0.0042 (8)	−0.0018 (8)	−0.0055 (9)
C14	0.0451 (11)	0.0527 (12)	0.0423 (11)	−0.0038 (9)	−0.0050 (9)	0.0002 (9)
C15	0.0498 (12)	0.0821 (16)	0.0573 (14)	0.0034 (11)	−0.0027 (10)	−0.0132 (12)
C16	0.0457 (12)	0.111 (2)	0.0716 (17)	−0.0057 (13)	−0.0079 (12)	−0.0113 (16)
C17	0.0582 (14)	0.103 (2)	0.0649 (15)	−0.0214 (13)	−0.0135 (12)	−0.0110 (15)
C18	0.0638 (14)	0.0689 (14)	0.0523 (13)	−0.0112 (11)	−0.0035 (11)	−0.0126 (11)
C19	0.0480 (11)	0.0495 (11)	0.0393 (10)	−0.0065 (9)	−0.0025 (9)	−0.0030 (9)
C20	0.0513 (12)	0.0477 (12)	0.0514 (12)	−0.0010 (9)	0.0001 (10)	−0.0042 (10)
N2A	0.087 (3)	0.074 (3)	0.0399 (19)	−0.015 (2)	−0.0150 (19)	0.0078 (18)
C21A	0.116 (5)	0.106 (4)	0.050 (3)	−0.020 (4)	−0.006 (3)	0.008 (3)
C22A	0.168 (6)	0.107 (6)	0.094 (6)	−0.040 (4)	0.016 (5)	−0.021 (4)
C23A	0.114 (4)	0.085 (4)	0.056 (3)	−0.028 (3)	−0.007 (3)	0.016 (3)
C24A	0.120 (7)	0.146 (9)	0.101 (3)	−0.052 (5)	0.003 (5)	−0.004 (5)
N2B	0.090 (6)	0.066 (5)	0.046 (4)	−0.022 (4)	−0.014 (4)	0.015 (4)
C21B	0.085 (7)	0.087 (10)	0.043 (6)	−0.032 (6)	0.006 (5)	−0.014 (5)
C22B	0.101 (6)	0.084 (6)	0.066 (5)	−0.021 (5)	−0.039 (5)	0.008 (4)
C23B	0.158 (16)	0.084 (8)	0.053 (5)	−0.037 (9)	−0.018 (9)	0.012 (6)
C24B	0.120 (7)	0.146 (9)	0.101 (3)	−0.052 (5)	0.003 (5)	−0.004 (5)
C25	0.124 (2)	0.0791 (19)	0.098 (2)	−0.0515 (19)	−0.0053 (19)	0.0119 (17)
C26	0.159 (3)	0.077 (2)	0.092 (2)	−0.027 (2)	−0.011 (2)	0.0052 (17)
C27	0.143 (3)	0.094 (2)	0.099 (2)	−0.049 (2)	−0.018 (2)	0.0329 (18)
C28A	0.146 (4)	0.089 (3)	0.110 (3)	−0.024 (3)	−0.024 (3)	0.005 (2)
C28B	0.161 (8)	0.109 (7)	0.098 (7)	−0.044 (7)	0.026 (6)	0.013 (6)
C29	0.0500 (11)	0.0600 (13)	0.0489 (12)	0.0002 (10)	0.0000 (9)	−0.0086 (10)
C30	0.0558 (12)	0.0769 (15)	0.0477 (12)	0.0038 (11)	0.0059 (10)	−0.0054 (11)
C31	0.0634 (13)	0.0677 (14)	0.0435 (12)	−0.0005 (11)	0.0020 (10)	0.0022 (11)
C32	0.0482 (11)	0.0532 (12)	0.0563 (13)	−0.0010 (9)	0.0014 (10)	0.0023 (10)
C33	0.0462 (11)	0.0538 (12)	0.0503 (12)	−0.0046 (9)	0.0071 (9)	−0.0020 (10)
C34	0.0518 (11)	0.0583 (13)	0.0468 (12)	0.0007 (10)	0.0009 (9)	−0.0010 (10)
C35	0.0577 (13)	0.0800 (16)	0.0551 (14)	0.0073 (12)	0.0006 (11)	−0.0075 (12)
C36	0.0872 (19)	0.120 (2)	0.088 (2)	0.0448 (17)	−0.0106 (16)	0.0104 (18)
C37	0.108 (2)	0.176 (3)	0.091 (2)	0.039 (2)	−0.019 (2)	0.011 (2)

### Geometric parameters (Å, °)

**Table:**

O1—C7	1.364 (2)	C22A—H22B	0.9600
O1—C5	1.371 (2)	C22A—H22C	0.9600
O2—C20	1.241 (2)	C23A—C24A	1.498 (6)
O3—C20	1.255 (2)	C23A—H23A	0.9700
O4—C31	1.371 (2)	C23A—H23B	0.9700
O4—H4O	0.8200	C24A—H24A	0.9600
O5—C32	1.352 (2)	C24A—H24B	0.9600
O5—H5O	0.8200	C24A—H24C	0.9600
O6—C33	1.354 (2)	N2B—C21B	1.471 (7)
O6—H6O	0.8200	N2B—C23B	1.478 (7)
O7—C35	1.204 (2)	C21B—C22B	1.513 (8)
O8—C35	1.315 (3)	C21B—H21C	0.9700
O8—C36	1.455 (3)	C21B—H21D	0.9700
N1—C1	1.350 (3)	C22B—H22D	0.9600
N1—C25	1.470 (3)	C22B—H22E	0.9600
N1—C27	1.479 (3)	C22B—H22F	0.9600
C1—C6	1.409 (3)	C23B—C24B	1.503 (9)
C1—C2	1.419 (3)	C23B—H23C	0.9700
C2—C3	1.351 (3)	C23B—H23D	0.9700
C2—H2	0.9300	C24B—H24D	0.9600
C3—C4	1.420 (3)	C24B—H24E	0.9600
C3—H3	0.9300	C24B—H24F	0.9600
C4—C13	1.394 (3)	C25—C26	1.490 (4)
C4—C5	1.405 (3)	C25—H25A	0.9700
C5—C6	1.366 (3)	C25—H25B	0.9700
C6—H6	0.9300	C26—H26A	0.9600
C7—C8	1.376 (3)	C26—H26B	0.9600
C7—C12	1.405 (3)	C26—H26C	0.9600
C8—C9	1.401 (3)	C27—C28B	1.415 (8)
C8—H8	0.9300	C27—C28A	1.422 (4)
C9—N2A	1.379 (4)	C27—H27A	0.9700
C9—N2B	1.423 (6)	C27—H27B	0.9700
C9—C10	1.424 (3)	C27—H27C	0.9700
C10—C11	1.350 (3)	C27—H27D	0.9700
C10—H10	0.9300	C28A—H28A	0.9600
C11—C12	1.413 (3)	C28A—H28B	0.9600
C11—H11	0.9300	C28A—H28C	0.9600
C12—C13	1.406 (3)	C28B—H28D	0.9600
C13—C14	1.502 (3)	C28B—H28E	0.9600
C14—C15	1.386 (3)	C28B—H28F	0.9600
C14—C19	1.393 (3)	C29—C34	1.381 (3)
C15—C16	1.376 (3)	C29—C30	1.386 (3)
C15—H15	0.9300	C29—C35	1.477 (3)
C16—C17	1.377 (3)	C30—C31	1.380 (3)
C16—H16	0.9300	C30—H30	0.9300
C17—C18	1.382 (3)	C31—C32	1.387 (3)
C17—H17	0.9300	C32—C33	1.400 (3)
C18—C19	1.388 (3)	C33—C34	1.385 (3)
C18—H18	0.9300	C34—H34	0.9300
C19—C20	1.506 (3)	C36—C37	1.435 (4)
N2A—C21A	1.465 (5)	C36—H36A	0.9700
N2A—C23A	1.467 (5)	C36—H36B	0.9700
C21A—C22A	1.509 (6)	C37—H37A	0.9600
C21A—H21A	0.9700	C37—H37B	0.9600
C21A—H21B	0.9700	C37—H37C	0.9600
C22A—H22A	0.9600		
			
O2···O6	2.5991 (18)	N2A···C28B^ii^	2.914 (11)
O3···O5	2.579 (2)	C22B···C22B^iii^	2.676 (12)
O4···O7^i^	2.811 (2)	C23A···C28B^ii^	2.504 (11)
O5···O3	2.579 (2)	C28B···N2A^ii^	2.914 (11)
O6···O2	2.5991 (18)	C28B···C23A^ii^	2.504 (11)
O7···O4^i^	2.811 (2)		
			
C7—O1—C5	120.40 (16)	N2B—C21B—C22B	121.2 (12)
C31—O4—H4O	109.5	N2B—C21B—H21C	107.0
C32—O5—H5O	109.5	C22B—C21B—H21C	107.0
C33—O6—H6O	109.5	N2B—C21B—H21D	107.0
C35—O8—C36	117.74 (19)	C22B—C21B—H21D	107.0
C1—N1—C25	121.1 (2)	H21C—C21B—H21D	106.8
C1—N1—C27	122.9 (2)	C21B—C22B—H22D	109.5
C25—N1—C27	115.9 (2)	C21B—C22B—H22E	109.5
N1—C1—C6	121.6 (2)	H22D—C22B—H22E	109.5
N1—C1—C2	120.8 (2)	C21B—C22B—H22F	109.5
C6—C1—C2	117.6 (2)	H22D—C22B—H22F	109.5
C3—C2—C1	121.5 (2)	H22E—C22B—H22F	109.5
C3—C2—H2	119.2	N2B—C23B—C24B	110.8 (8)
C1—C2—H2	119.2	N2B—C23B—H23C	109.5
C2—C3—C4	121.9 (2)	C24B—C23B—H23C	109.5
C2—C3—H3	119.0	N2B—C23B—H23D	109.5
C4—C3—H3	119.0	C24B—C23B—H23D	109.5
C13—C4—C5	120.04 (17)	H23C—C23B—H23D	108.1
C13—C4—C3	124.57 (19)	C23B—C24B—H24D	109.5
C5—C4—C3	115.39 (18)	C23B—C24B—H24E	109.5
C6—C5—O1	115.64 (18)	H24D—C24B—H24E	109.5
C6—C5—C4	123.93 (18)	C23B—C24B—H24F	109.5
O1—C5—C4	120.37 (17)	H24D—C24B—H24F	109.5
C5—C6—C1	119.5 (2)	H24E—C24B—H24F	109.5
C5—C6—H6	120.3	N1—C25—C26	113.4 (2)
C1—C6—H6	120.3	N1—C25—H25A	108.9
O1—C7—C8	115.50 (18)	C26—C25—H25A	108.9
O1—C7—C12	120.75 (17)	N1—C25—H25B	108.9
C8—C7—C12	123.68 (19)	C26—C25—H25B	108.9
C7—C8—C9	119.1 (2)	H25A—C25—H25B	107.7
C7—C8—H8	120.4	C25—C26—H26A	109.5
C9—C8—H8	120.4	C25—C26—H26B	109.5
N2A—C9—C8	123.0 (3)	H26A—C26—H26B	109.5
C8—C9—N2B	116.2 (3)	C25—C26—H26C	109.5
N2A—C9—C10	118.2 (3)	H26A—C26—H26C	109.5
C8—C9—C10	118.1 (2)	H26B—C26—H26C	109.5
N2B—C9—C10	120.7 (4)	C28B—C27—C28A	127.0 (6)
C11—C10—C9	121.2 (2)	C28B—C27—N1	114.1 (6)
C11—C10—H10	119.4	C28A—C27—N1	116.6 (3)
C9—C10—H10	119.4	C28B—C27—H27A	68.6
C10—C11—C12	122.0 (2)	C28A—C27—H27A	108.1
C10—C11—H11	119.0	N1—C27—H27A	108.1
C12—C11—H11	119.0	C28A—C27—H27B	108.1
C7—C12—C13	119.51 (18)	N1—C27—H27B	108.1
C7—C12—C11	115.67 (18)	H27A—C27—H27B	107.3
C13—C12—C11	124.82 (19)	C28B—C27—H27C	108.7
C4—C13—C12	118.90 (18)	N1—C27—H27C	108.7
C4—C13—C14	120.76 (16)	H27A—C27—H27C	140.0
C12—C13—C14	119.77 (17)	H27B—C27—H27C	74.9
C15—C14—C19	119.29 (19)	C28B—C27—H27D	108.7
C15—C14—C13	116.78 (18)	C28A—C27—H27D	68.4
C19—C14—C13	123.91 (16)	N1—C27—H27D	108.7
C16—C15—C14	121.2 (2)	H27B—C27—H27D	139.7
C16—C15—H15	119.4	H27C—C27—H27D	107.6
C14—C15—H15	119.4	C27—C28A—H28A	109.5
C15—C16—C17	119.9 (2)	C27—C28A—H28B	109.5
C15—C16—H16	120.1	H28A—C28A—H28B	109.5
C17—C16—H16	120.1	C27—C28A—H28C	109.5
C16—C17—C18	119.3 (2)	H28A—C28A—H28C	109.5
C16—C17—H17	120.3	H28B—C28A—H28C	109.5
C18—C17—H17	120.3	C27—C28B—H28D	109.5
C17—C18—C19	121.4 (2)	C27—C28B—H28E	109.5
C17—C18—H18	119.3	H28D—C28B—H28E	109.5
C19—C18—H18	119.3	C27—C28B—H28F	109.5
C18—C19—C14	118.84 (18)	H28D—C28B—H28F	109.5
C18—C19—C20	119.97 (18)	H28E—C28B—H28F	109.5
C14—C19—C20	121.18 (18)	C34—C29—C30	119.53 (19)
O2—C20—O3	124.83 (19)	C34—C29—C35	122.0 (2)
O2—C20—C19	118.03 (18)	C30—C29—C35	118.48 (19)
O3—C20—C19	117.13 (19)	C31—C30—C29	119.74 (19)
C9—N2A—C21A	119.5 (4)	C31—C30—H30	120.1
C9—N2A—C23A	122.6 (4)	C29—C30—H30	120.1
C21A—N2A—C23A	117.2 (4)	O4—C31—C30	122.02 (19)
N2A—C21A—C22A	110.3 (7)	O4—C31—C32	116.56 (19)
N2A—C21A—H21A	109.6	C30—C31—C32	121.4 (2)
C22A—C21A—H21A	109.6	O5—C32—C31	118.64 (19)
N2A—C21A—H21B	109.6	O5—C32—C33	122.70 (18)
C22A—C21A—H21B	109.6	C31—C32—C33	118.55 (18)
H21A—C21A—H21B	108.1	O6—C33—C34	116.86 (18)
C21A—C22A—H22A	109.5	O6—C33—C32	123.40 (18)
C21A—C22A—H22B	109.5	C34—C33—C32	119.75 (18)
H22A—C22A—H22B	109.5	C29—C34—C33	120.9 (2)
C21A—C22A—H22C	109.5	C29—C34—H34	119.5
H22A—C22A—H22C	109.5	C33—C34—H34	119.5
H22B—C22A—H22C	109.5	O7—C35—O8	122.1 (2)
N2A—C23A—C24A	113.7 (5)	O7—C35—C29	124.9 (2)
N2A—C23A—H23A	108.8	O8—C35—C29	112.99 (19)
C24A—C23A—H23A	108.8	C37—C36—O8	108.5 (2)
N2A—C23A—H23B	108.8	C37—C36—H36A	110.0
C24A—C23A—H23B	108.8	O8—C36—H36A	110.0
H23A—C23A—H23B	107.7	C37—C36—H36B	110.0
C23A—C24A—H24A	109.5	O8—C36—H36B	110.0
C23A—C24A—H24B	109.5	H36A—C36—H36B	108.4
H24A—C24A—H24B	109.5	C36—C37—H37A	109.5
C23A—C24A—H24C	109.5	C36—C37—H37B	109.5
H24A—C24A—H24C	109.5	H37A—C37—H37B	109.5
H24B—C24A—H24C	109.5	C36—C37—H37C	109.5
C9—N2B—C21B	124.8 (7)	H37A—C37—H37C	109.5
C9—N2B—C23B	120.8 (7)	H37B—C37—H37C	109.5
C21B—N2B—C23B	113.6 (8)		

Symmetry codes: (i) −*x*, −*y*+2, −*z*+2; (ii) −*x*+1, −*y*+1, −*z*+1; (iii) −*x*+1, −*y*+1, −*z*.

### Hydrogen-bond geometry (Å, °)

**Table:**

*D*—H···*A*	*D*—H	H···*A*	*D*···*A*	*D*—H···*A*
O4—H4O···O7^i^	0.82	2.00	2.811 (2)	168
O5—H5O···O2	0.82	2.79	3.257 (2)	118
O5—H5O···O3	0.82	1.78	2.579 (2)	164
O6—H6O···O2	0.82	1.86	2.5991 (18)	148
O6—H6O···O3	0.82	2.78	3.3842 (19)	132
O6—H6O···O5	0.82	2.47	2.8758 (19)	112

Symmetry codes: (i) −*x*, −*y*+2, −*z*+2.
